# The prognostic value of cardiovascular magnetic resonance in aborted sudden cardiac death

**DOI:** 10.1186/1532-429X-17-S1-O31

**Published:** 2015-02-03

**Authors:** Peter P Swoboda, Ananth Kidambi, John Gierula, Adam K McDiarmid, Bara Erhayiem, Laura E Dobson, Tarique A Musa, David P Ripley, Pankaj Garg, Klaus K Witte, John P Greenwood, Sven Plein

**Affiliations:** 1 Multidisciplinary Cardiovascular Research Centre & Leeds Institute of Cardiovascular and Metabolic Medicine, University of Leeds, Leeds, UK

## Background

Aborted sudden cardiac death (SCD) is a common indication for CMR. No studies have investigated the prognostic value of late gadolinium enhancement (LGE) solely in patients with secondary prevention implantable cardioverter-defibrillators (ICD) or aborted SCD.

## Methods

58 consecutive patients who had CMR and ICD implantation after aborted SCD between 2006 and 2013 were identified. Clinical notes and CMR results were reviewed. Patients were scanned with a CMR protocol that included a short axis cine stack and LGE imaging. CMR images were analysed blinded to clinical data using standard software (cvi42, Circle CVI, Canada). Patients were followed up after ICD implantation at six weeks, three months and six months and then every six months. Appropriate ICD therapy was defined as any anti-tachycardia pacing (ATP) or shock therapy delivered for a ventricular arrhythmia.

## Results

Follow up data was available on 55/58 patients. Four patients died during the follow up period and one patient was lost to follow up 14 days post-implantation. Median follow up was 791 days (interquartile range 370-1142 days). 11 patients had appropriate ICD therapy. 6 had ATP only and 5 had appropriate shocks ±ATP for ventricular arrhythmias. The clinical aetiology of the aborted SCD was ischaemic heart disease (IHD) 24, primary arrhythmia 14, arrhythmogenic right ventricular cardiomyopathy 7, dilated cardiomyopathy 6, myocarditis 2 and other 5.

**Figure 1:**
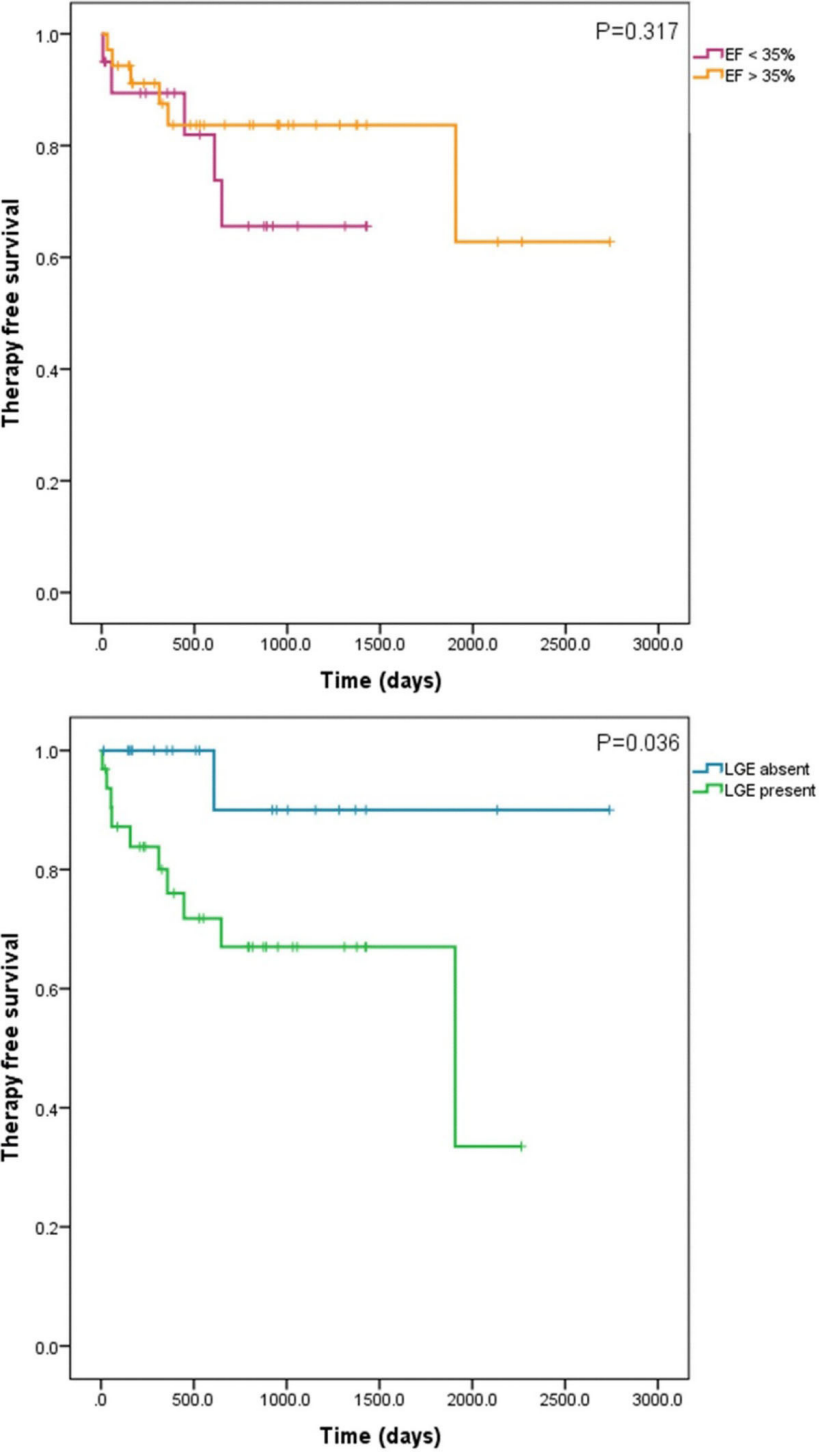
Kaplan Meier curves showing freedom from appropriate ICD therapy with patients stratified according to presence of LGE (above) and EF (below). Each horizontal line represents a censored subject.

Of 55 patients on whom clinical outcome data was available 20 patients had ejection fraction (EF)<35% and 35 had EF>35%. 5/20 (25%) of those with EF<35% had appropriate ICD therapies and 6/35 (17%) of those with EF>35% had appropriate ICD therapies on follow up. There was no significant difference in the likelihood ratio (LR) of appropriate therapy using an EF cutoff of 35% (LR 0.48, P=0.49), confirmed on log rank analysis of Kaplan Meier curves (figure 1) (P=0.32).

Of the 52 patients who had LGE imaging and clinical outcome data 32 had LGE present and 20 had no LGE. 10/32 (31%) LGE +ve patients had appropriate ICD therapies on follow up and only 1/20 (5%) LGE-ve patients had appropriate ICD therapies. LGE +ve patients were significantly more likely to have an appropriate ICD therapy on follow up (LR 5.97, P=0.015), confirmed on log rank analysis of Kaplan Meier curves (P=0.036).

## Conclusions

In patients who have suffered aborted SCD, CMR is able to identify patients at risk of future arrhythmic events according to the presence or absence of LGE.

## Funding

PPS (FS/12/88/29901) and SP (FS/10/62/28409) are funded by British Heart Foundation fellowships.

